# [1,2-Bis(diphenyl­phosphino)ethane]{2-[bis­(diphenyl­phosphinometh­yl)amino]pyridinium}fluoridohydrazidato­molybdenum(IV) bis­(tetra­fluoridoborate)

**DOI:** 10.1107/S1600536808008635

**Published:** 2008-04-10

**Authors:** Gerald Stephan, Christian Näther, Felix Tuczek

**Affiliations:** a Institut für Anorganische Chemie, Christian-Albrechts-Universität Kiel, Olshausenstrasse 40, D-24098 Kiel, Germany

## Abstract

In the crystal structure of the title compound, [MoF(N_2_H_2_)(C_31_H_29_N_2_P_2_)(C_26_H_24_P_2_)](BF_4_)_2_, each Mo atom is surrounded by four P atoms of one 1,2-bis­(diphenyl­phosphino)ethane and one 2-[bis­(diphenyl­phosphinometh­yl)amino]pyridinium ligand. The remaining binding sites of the distorted octa­hedron are occupied by a hydrazidate (NNH_2_^2−^) and a fluoride ligand. Two F atoms of an anion are disordered over two positions; the site occupancy factors are *ca* 0.7 and 0.3.

## Related literature

For related literature, see: Hidai *et al.* (1976 ▶); Stephan *et al.* (2008 ▶).
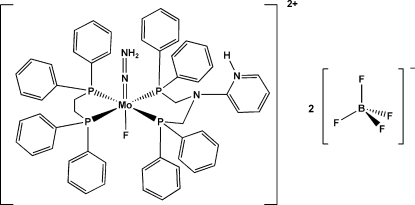


## Experimental

### 

#### Crystal data

[MoF(N_2_H_2_)(C_31_H_29_N_2_P_2_)(C_26_H_24_P_2_)](BF_4_)_2_
*M* *_r_* = 1208.49
Monoclinic, 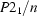

*a* = 12.5791 (6) Å
*b* = 31.2199 (16) Å
*c* = 13.8330 (9) Å
β = 94.438 (7)°
*V* = 5416.2 (5) Å^3^
*Z* = 4
Mo *K*α radiation
μ = 0.43 mm^−1^
*T* = 170 (2) K
0.2 × 0.08 × 0.06 mm

#### Data collection

Stoe IPDS-1 diffractometer
Absorption correction: numerical (*X-SHAPE*; Stoe & Cie, 1998*a* ▶) *T*_min_ = 0.949, *T*_max_ = 0.969
24176 measured reflections
8538 independent reflections
6783 reflections with *I* > 2σ(*I*)
*R*_int_ = 0.032
θ_max_ = 24.1°

#### Refinement

*R*[*F* ^2^ > 2σ(*F* ^2^)] = 0.039
*wR*(*F* ^2^) = 0.108
*S* = 1.02
8538 reflections
706 parameters
39 restraints
H-atom parameters constrained
Δρ_max_ = 0.94 e Å^−3^
Δρ_min_ = −0.54 e Å^−3^

### 

Data collection: *IPDS Program package* (Stoe & Cie, 1998*b* ▶); cell refinement: *IPDS Program package*; data reduction: *IPDS Program package*; program(s) used to solve structure: *SHELXS97* (Sheldrick, 2008 ▶); program(s) used to refine structure: *SHELXL97* (Sheldrick, 2008 ▶); molecular graphics: *DIAMOND* (Brandenburg, 1999 ▶); software used to prepare material for publication: *CIFTAB* in *SHELXTL* (Sheldrick, 2008 ▶).

## Supplementary Material

Crystal structure: contains datablocks I, global. DOI: 10.1107/S1600536808008635/bt2688sup1.cif

Structure factors: contains datablocks I. DOI: 10.1107/S1600536808008635/bt2688Isup2.hkl

Additional supplementary materials:  crystallographic information; 3D view; checkCIF report

## Figures and Tables

**Table:** 

Mo1—N1	1.755 (3)
Mo1—F1	1.9889 (18)
Mo1—P1	2.5032 (9)
Mo1—P2	2.5353 (9)
Mo1—P3	2.5698 (8)
Mo1—P4	2.5754 (9)

**Table:** 

N1—Mo1—F1	178.70 (11)
N1—Mo1—P1	86.88 (10)
F1—Mo1—P1	91.94 (6)
N1—Mo1—P2	86.22 (9)
F1—Mo1—P2	94.33 (6)
P1—Mo1—P2	89.65 (3)
N1—Mo1—P3	94.46 (9)
F1—Mo1—P3	85.10 (5)
P1—Mo1—P3	95.38 (3)
P2—Mo1—P3	174.95 (3)
N1—Mo1—P4	101.62 (10)
F1—Mo1—P4	79.52 (6)
P1—Mo1—P4	170.35 (3)
P2—Mo1—P4	95.42 (3)
P3—Mo1—P4	79.53 (3)
N2—N1—Mo1	177.1 (3)

**Fig. 1.**
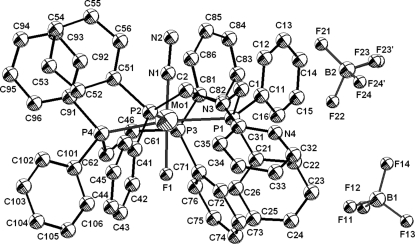
: Crystal structure of the title compound with labelling and displacement ellipsoids drawn at the 50% probability level. The H atoms are omitted for clarity.

## supplementary crystallographic information

### Comment

The structure determination of this compound was undertaken as part of a project on nitrogen fixation on molybdenum(0) centers with PNP co-ligands. The compound was synthesized by protonation of the Mo(0) complex [Mo(N_2_)_2_(dppe)(pyNP_2_)] (Stephan *et al.*, 2008) with HBF_4_.Et_2_O in analogy to the known procedure (Hidai *et al.*, 1976). Beside the double protonation of one dinitrogen ligand the complex is secondary protonated at the pyridine N atom of the pyNP_2_ ligand. The second N_2_ ligand is exchanged against a fluoride ligand during the protonation.

The crystal structure of the title compound consists of discrete [Mo(F)(NNH_2_)(P_2_C_26_H_24_)(P_2_C_31_H_29_N_2_)]^2+^ cations and tetrafluoroborate anions. Each molybdenum atom is coordinated by four P atoms of one 1,2-bis(diphenylphosphino)ethane and one *N*,*N*-bis(diphenylphosphinomethyl)-2-aminopyridinium ligand, one N atom a hydrazido ligand and a fluoro ligand within a distorted octahetral geometry. The bite angles of the phosphine ligands are 79.53 (2)° for the dppe ligand and 89.66 (2)° for the pyHNP_2_ ligand. The Mo—N distance is 1.755 (2)Å and the N—N distance is 1.326 (4) Å.

### Experimental

Under an inert gas atmosphere 0.115 ml HBF_4_*Et_2_O were added to 300 mg of [Mo(N_2_)_2_(dppe)(pyNP_2_)] in 8 ml Dichloromethane at -15°C. The brown solution was stirred for 15 min and 15 ml of n-Hexane were added. The brown solid was filtered off, washed four times with diethylether and dried under vacuum. Yield: 270 mg (82%). Single crystals were obtained by diffusion of diethylether into a methanolic solution of the complex over a period of one week. ^31^P NMR (CD_2_Cl_2_, 20°C): 43.5 (2 P, dppe), 16.9 (2 P, pyHNP_2_), ^2^J_PP,_*_trans_*= 132.0 Hz (P1—P4, P2—P4), ^2^J_PP,_*_cis_*= -26.5 Hz (P2—P3, P1—P4), ^2^J_PP,_*_cis_*= -33.0 Hz (P1—P2), ^2^J_PP,_*_cis_*= 3.0 Hz (P3—P4), ^2^J_PF,_*_cis_*= -31.0 Hz. ^19^F NMR (CD_2_Cl_2_, 20°C) 148.1 (Mo—F), q, 31 Hz), 149.5 (BF_4_^-^, s)

### Refinement

The C—H H atoms and the N—H H atom of the 6-membered ring were positioned with idealized geometry and refined using a riding model with *U*_iso_(H)=1.2*U*_eq_(carrier atom) and with C—H = 0.95–0.99 Å and N—H = 0.88 Å or N—H= 0.99 Å. Two of the four F atoms of the tetrafluoroborate anion are disordered over two positions with site occupation factors of 0.737 (9) and 0.263 (9). The minor occupied sites were refined isotropically. The bond lengths and angles of the disordered BF_4_ anion were restrained to be equal to those of the not disordered one.

### Crystal data

**Table:**

[MoF(N_2_H_2_)(C_31_H_29_N_2_P_2_)(C_26_H_24_P_2_)](BF_4_)_2_	*F*_000_ = 2472
*M**_r_* = 1208.49	*D*_x_ = 1.482 Mg m^−^^3^
Monoclinic, *P*2_1_/*n*	Mo *K*α radiation λ = 0.71073 Å
*a* = 12.5791 (6) Å	Cell parameters from 8000 reflections
*b* = 31.2199 (16) Å	θ = 10–22º
*c* = 13.8330 (9) Å	µ = 0.44 mm^−^^1^
β = 94.438 (7)º	*T* = 170 (2) K
*V* = 5416.2 (5) Å^3^	Needle, colourless
*Z* = 4	0.2 × 0.08 × 0.06 mm

### Data collection

**Table:**

Stoe IPDS-1 diffractometer	8538 independent reflections
Radiation source: fine-focus sealed tube	6783 reflections with *I* > 2σ(*I*)
Monochromator: graphite	*R*_int_ = 0.032
*T* = 170(2) K	θ_max_ = 24.1º
φ scans	θ_min_ = 1.8º
Absorption correction: numerical(X-SHAPE; Stoe & Cie, 1998a)	*h* = −14→14
*T*_min_ = 0.949, *T*_max_ = 0.969	*k* = −35→35
24176 measured reflections	*l* = −15→15

### Refinement

**Table:**

Refinement on *F*^2^	Secondary atom site location: difference Fourier map
Least-squares matrix: full	Hydrogen site location: inferred from neighbouring sites
*R*[*F*^2^ > 2σ(*F*^2^)] = 0.039	H-atom parameters constrained
*wR*(*F*^2^) = 0.108	*w* = 1/[σ^2^(*F*_o_^2^) + (0.0748*P*)^2^] where *P* = (*F*_o_^2^ + 2*F*_c_^2^)/3
*S* = 1.02	(Δ/σ)_max_ = 0.001
8538 reflections	Δρ_max_ = 0.94 e Å^−^^3^
706 parameters	Δρ_min_ = −0.54 e Å^−^^3^
39 restraints	Extinction correction: SHELXL97 (Sheldrick, 2008), Fc^*^=kFc[1+0.001xFc^2^λ^3^/sin(2θ)]^-1/4^
Primary atom site location: structure-invariant direct methods	Extinction coefficient: 0.0045 (4)

### Special details

**Table:**

Geometry. All e.s.d.'s (except the e.s.d. in the dihedral angle between two l.s. planes) are estimated using the full covariance matrix. The cell e.s.d.'s are taken into account individually in the estimation of e.s.d.'s in distances, angles and torsion angles; correlations between e.s.d.'s in cell parameters are only used when they are defined by crystal symmetry. An approximate (isotropic) treatment of cell e.s.d.'s is used for estimating e.s.d.'s involving l.s. planes.
Refinement. Refinement of *F*^2^ against ALL reflections. The weighted *R*-factor *wR* and goodness of fit *S* are based on *F*^2^, conventional *R*-factors *R* are based on *F*, with *F* set to zero for negative *F*^2^. The threshold expression of *F*^2^ > σ(*F*^2^) is used only for calculating *R*-factors(gt) *etc*. and is not relevant to the choice of reflections for refinement. *R*-factors based on *F*^2^ are statistically about twice as large as those based on *F*, and *R*- factors based on ALL data will be even larger.

### Fractional atomic coordinates and isotropic or equivalent isotropic displacement parameters (Å^2^)

**Table:**

	*x*	*y*	*z*	*U*_iso_*/*U*_eq_	Occ. (<1)
Mo1	0.10191 (2)	0.148150 (8)	0.284941 (19)	0.01774 (12)	
F1	0.20855 (15)	0.17169 (6)	0.20023 (13)	0.0234 (4)	
N1	0.0094 (2)	0.12792 (9)	0.3620 (2)	0.0251 (6)	
N2	−0.0567 (5)	0.11290 (17)	0.4239 (3)	0.094 (2)	
H1N2	−0.0661	0.1300	0.4759	0.13 (3)*	
H2N2	−0.0890	0.0868	0.4206	0.11 (2)*	
C1	0.2510 (3)	0.09669 (10)	0.4909 (2)	0.0232 (7)	
H1A	0.3011	0.0995	0.5493	0.028*	
H1B	0.1817	0.0866	0.5117	0.028*	
N3	0.2925 (2)	0.06529 (8)	0.4248 (2)	0.0250 (6)	
C2	0.2109 (3)	0.04163 (10)	0.3656 (2)	0.0261 (8)	
H2A	0.1529	0.0339	0.4068	0.031*	
H2B	0.2427	0.0146	0.3440	0.031*	
P1	0.23297 (7)	0.14991 (2)	0.43059 (6)	0.0184 (2)	
P2	0.15116 (7)	0.07070 (3)	0.25607 (6)	0.0205 (2)	
C11	0.1921 (3)	0.18206 (10)	0.5315 (2)	0.0205 (7)	
C12	0.1252 (3)	0.16457 (12)	0.5967 (3)	0.0301 (8)	
H12	0.1071	0.1351	0.5928	0.036*	
C13	0.0849 (3)	0.19017 (13)	0.6676 (3)	0.0376 (10)	
H13	0.0390	0.1780	0.7116	0.045*	
C14	0.1109 (3)	0.23297 (12)	0.6747 (3)	0.0331 (9)	
H14	0.0832	0.2502	0.7234	0.040*	
C15	0.1775 (3)	0.25078 (11)	0.6104 (3)	0.0289 (8)	
H15	0.1961	0.2802	0.6154	0.035*	
C16	0.2171 (3)	0.22555 (11)	0.5385 (2)	0.0249 (7)	
H16	0.2617	0.2380	0.4938	0.030*	
C21	0.3723 (3)	0.16364 (10)	0.4168 (2)	0.0199 (7)	
C22	0.4412 (3)	0.17702 (11)	0.4954 (3)	0.0282 (8)	
H22	0.4140	0.1820	0.5566	0.034*	
C23	0.5490 (3)	0.18301 (13)	0.4845 (3)	0.0391 (10)	
H23	0.5953	0.1919	0.5383	0.047*	
C24	0.5890 (3)	0.17612 (14)	0.3955 (3)	0.0431 (10)	
H24	0.6627	0.1803	0.3879	0.052*	
C25	0.5212 (3)	0.16314 (14)	0.3172 (3)	0.0387 (10)	
H25	0.5489	0.1584	0.2562	0.046*	
C26	0.4132 (3)	0.15695 (11)	0.3272 (2)	0.0279 (8)	
H26	0.3674	0.1482	0.2730	0.034*	
C31	0.3978 (3)	0.05601 (11)	0.4229 (3)	0.0275 (8)	
N4	0.4696 (3)	0.07235 (9)	0.4921 (2)	0.0323 (7)	
H4	0.4453	0.0885	0.5377	0.039*	
C32	0.5761 (3)	0.06524 (14)	0.4949 (3)	0.0443 (10)	
H32	0.6221	0.0780	0.5445	0.053*	
C33	0.6175 (4)	0.04001 (15)	0.4273 (4)	0.0520 (12)	
H33	0.6920	0.0348	0.4287	0.062*	
C34	0.5476 (4)	0.02198 (13)	0.3558 (3)	0.0477 (11)	
H34	0.5751	0.0041	0.3081	0.057*	
C35	0.4399 (3)	0.02946 (12)	0.3524 (3)	0.0382 (9)	
H35	0.3937	0.0168	0.3028	0.046*	
C41	0.2374 (3)	0.05482 (11)	0.1619 (2)	0.0248 (7)	
C42	0.2806 (3)	0.08551 (12)	0.1042 (3)	0.0279 (8)	
H42	0.2661	0.1150	0.1141	0.034*	
C43	0.3453 (3)	0.07327 (13)	0.0314 (3)	0.0348 (9)	
H43	0.3731	0.0944	−0.0091	0.042*	
C44	0.3689 (3)	0.03079 (14)	0.0183 (3)	0.0385 (10)	
H44	0.4139	0.0227	−0.0307	0.046*	
C45	0.3275 (3)	−0.00026 (13)	0.0759 (3)	0.0392 (10)	
H45	0.3447	−0.0296	0.0670	0.047*	
C46	0.2603 (3)	0.01148 (12)	0.1473 (3)	0.0337 (9)	
H46	0.2303	−0.0099	0.1858	0.040*	
C51	0.0310 (3)	0.03842 (10)	0.2289 (2)	0.0242 (7)	
C52	−0.0067 (3)	0.03302 (11)	0.1324 (3)	0.0304 (8)	
H52	0.0346	0.0432	0.0825	0.037*	
C53	−0.1037 (3)	0.01295 (13)	0.1077 (3)	0.0388 (9)	
H53	−0.1286	0.0098	0.0415	0.047*	
C54	−0.1641 (3)	−0.00240 (12)	0.1797 (3)	0.0391 (10)	
H54	−0.2308	−0.0158	0.1631	0.047*	
C55	−0.1271 (3)	0.00183 (13)	0.2755 (3)	0.0382 (9)	
H55	−0.1678	−0.0093	0.3249	0.046*	
C56	−0.0302 (3)	0.02239 (12)	0.3006 (3)	0.0337 (9)	
H56	−0.0058	0.0255	0.3670	0.040*	
C61	−0.0634 (3)	0.23553 (11)	0.2013 (2)	0.0263 (8)	
H61A	−0.1315	0.2229	0.2191	0.032*	
H61B	−0.0743	0.2667	0.1916	0.032*	
C62	−0.0315 (3)	0.21521 (11)	0.1075 (2)	0.0260 (8)	
H62A	0.0361	0.2280	0.0892	0.031*	
H62B	−0.0872	0.2208	0.0545	0.031*	
P3	0.04148 (7)	0.22633 (3)	0.30016 (6)	0.0194 (2)	
P4	−0.01447 (7)	0.15685 (3)	0.12462 (6)	0.0210 (2)	
C71	0.1408 (3)	0.26775 (10)	0.2819 (2)	0.0219 (7)	
C72	0.2489 (3)	0.25795 (11)	0.2986 (2)	0.0240 (7)	
H72	0.2701	0.2295	0.3154	0.029*	
C73	0.3259 (3)	0.28937 (11)	0.2910 (3)	0.0297 (8)	
H73	0.3991	0.2824	0.3038	0.036*	
C74	0.2969 (3)	0.33074 (12)	0.2649 (3)	0.0334 (9)	
H74	0.3498	0.3521	0.2592	0.040*	
C75	0.1905 (3)	0.34069 (11)	0.2474 (3)	0.0337 (9)	
H75	0.1704	0.3691	0.2296	0.040*	
C76	0.1119 (3)	0.30973 (11)	0.2552 (3)	0.0288 (8)	
H76	0.0388	0.3171	0.2425	0.035*	
C81	−0.0282 (3)	0.24415 (11)	0.4045 (2)	0.0238 (7)	
C82	−0.0130 (3)	0.28421 (12)	0.4469 (3)	0.0380 (9)	
H82	0.0382	0.3033	0.4238	0.046*	
C83	−0.0724 (4)	0.29663 (14)	0.5233 (3)	0.0476 (11)	
H83	−0.0632	0.3245	0.5501	0.057*	
C84	−0.1438 (3)	0.26910 (15)	0.5600 (3)	0.0453 (11)	
H84	−0.1829	0.2776	0.6129	0.054*	
C85	−0.1585 (4)	0.22907 (16)	0.5197 (3)	0.0510 (12)	
H85	−0.2075	0.2097	0.5453	0.061*	
C86	−0.1020 (3)	0.21664 (14)	0.4415 (3)	0.0441 (11)	
H86	−0.1140	0.1892	0.4133	0.053*	
C91	−0.1518 (3)	0.13741 (11)	0.1137 (3)	0.0252 (8)	
C92	−0.1915 (3)	0.11545 (12)	0.1905 (3)	0.0342 (9)	
H92	−0.1479	0.1114	0.2490	0.041*	
C93	−0.2950 (3)	0.09930 (14)	0.1819 (3)	0.0464 (11)	
H93	−0.3218	0.0846	0.2350	0.056*	
C94	−0.3590 (3)	0.10451 (14)	0.0970 (4)	0.0488 (12)	
H94	−0.4291	0.0930	0.0913	0.059*	
C95	−0.3208 (3)	0.12639 (13)	0.0209 (3)	0.0428 (10)	
H95	−0.3649	0.1301	−0.0374	0.051*	
C96	−0.2178 (3)	0.14327 (12)	0.0284 (3)	0.0323 (9)	
H96	−0.1925	0.1588	−0.0243	0.039*	
C101	0.0390 (3)	0.14118 (11)	0.0102 (2)	0.0259 (8)	
C102	−0.0050 (3)	0.10789 (12)	−0.0474 (2)	0.0315 (8)	
H102	−0.0669	0.0935	−0.0293	0.038*	
C103	0.0417 (4)	0.09577 (13)	−0.1312 (3)	0.0426 (10)	
H103	0.0106	0.0735	−0.1707	0.051*	
C104	0.1324 (4)	0.11582 (15)	−0.1572 (3)	0.0486 (12)	
H104	0.1652	0.1068	−0.2133	0.058*	
C105	0.1758 (4)	0.14918 (15)	−0.1015 (3)	0.0462 (11)	
H105	0.2377	0.1634	−0.1205	0.055*	
C106	0.1299 (3)	0.16213 (13)	−0.0178 (3)	0.0348 (9)	
H106	0.1602	0.1851	0.0200	0.042*	
B1	0.8583 (5)	0.07358 (16)	0.6061 (4)	0.0541 (15)	
F11	0.8620 (2)	0.04255 (7)	0.53264 (17)	0.0507 (6)	
F12	0.8016 (3)	0.10857 (9)	0.5658 (2)	0.0746 (9)	
F13	0.9582 (3)	0.08731 (11)	0.6351 (2)	0.0925 (12)	
F14	0.8083 (4)	0.05738 (10)	0.6817 (2)	0.1209 (19)	
B2	0.4315 (4)	0.09191 (16)	0.7490 (3)	0.0497 (14)	
F21	0.3459 (3)	0.06624 (9)	0.7323 (3)	0.0867 (12)	
F22	0.4582 (3)	0.11052 (11)	0.66855 (19)	0.0921 (13)	
F23	0.4439 (5)	0.11424 (17)	0.8310 (3)	0.099 (3)	0.737 (9)
F24	0.5265 (4)	0.06407 (15)	0.7551 (6)	0.127 (3)	0.737 (9)
F23'	0.3739 (10)	0.1356 (3)	0.7658 (10)	0.080 (5)*	0.263 (9)
F24'	0.4862 (11)	0.0855 (5)	0.8262 (9)	0.104 (6)*	0.263 (9)

### Atomic displacement parameters (Å^2^)

**Table:**

	*U*^11^	*U*^22^	*U*^33^	*U*^12^	*U*^13^	*U*^23^
Mo1	0.02013 (18)	0.01895 (17)	0.01402 (17)	−0.00276 (11)	0.00048 (11)	0.00024 (10)
F1	0.0252 (11)	0.0248 (9)	0.0203 (10)	−0.0029 (8)	0.0036 (8)	0.0021 (7)
N1	0.0319 (17)	0.0252 (14)	0.0188 (14)	−0.0091 (13)	0.0066 (12)	−0.0024 (11)
N2	0.137 (5)	0.091 (4)	0.063 (3)	−0.066 (3)	0.062 (3)	−0.020 (3)
C1	0.027 (2)	0.0224 (17)	0.0201 (17)	−0.0012 (14)	0.0023 (14)	0.0033 (13)
N3	0.0283 (18)	0.0211 (14)	0.0250 (16)	−0.0007 (12)	−0.0015 (12)	−0.0007 (11)
C2	0.031 (2)	0.0235 (16)	0.0233 (18)	−0.0019 (15)	−0.0013 (15)	0.0011 (14)
P1	0.0201 (5)	0.0209 (4)	0.0141 (4)	−0.0019 (3)	0.0004 (3)	0.0007 (3)
P2	0.0250 (5)	0.0196 (4)	0.0169 (4)	−0.0027 (3)	0.0016 (3)	−0.0010 (3)
C11	0.0166 (17)	0.0289 (17)	0.0155 (16)	0.0003 (14)	−0.0027 (13)	−0.0003 (13)
C12	0.030 (2)	0.0307 (18)	0.030 (2)	0.0013 (16)	0.0106 (16)	0.0008 (15)
C13	0.039 (2)	0.045 (2)	0.031 (2)	0.0063 (18)	0.0168 (18)	0.0038 (17)
C14	0.035 (2)	0.036 (2)	0.029 (2)	0.0121 (17)	0.0058 (17)	−0.0061 (16)
C15	0.029 (2)	0.0280 (18)	0.029 (2)	0.0034 (15)	−0.0028 (16)	−0.0059 (15)
C16	0.0246 (19)	0.0280 (18)	0.0220 (18)	−0.0012 (14)	0.0016 (14)	−0.0010 (14)
C21	0.0189 (18)	0.0209 (15)	0.0200 (17)	0.0010 (13)	0.0009 (13)	0.0017 (13)
C22	0.024 (2)	0.0356 (19)	0.0248 (19)	−0.0012 (15)	0.0017 (15)	−0.0049 (15)
C23	0.025 (2)	0.050 (2)	0.041 (2)	−0.0065 (18)	−0.0040 (17)	−0.0108 (19)
C24	0.025 (2)	0.061 (3)	0.045 (3)	−0.0055 (19)	0.0092 (19)	−0.004 (2)
C25	0.031 (2)	0.056 (2)	0.030 (2)	0.0044 (19)	0.0115 (18)	−0.0018 (18)
C26	0.027 (2)	0.037 (2)	0.0198 (18)	0.0027 (16)	0.0016 (15)	0.0005 (15)
C31	0.031 (2)	0.0244 (17)	0.0263 (19)	0.0024 (15)	−0.0007 (15)	0.0067 (14)
N4	0.0311 (19)	0.0309 (16)	0.0340 (18)	0.0045 (14)	−0.0024 (14)	0.0025 (13)
C32	0.032 (2)	0.047 (2)	0.052 (3)	0.0032 (19)	−0.008 (2)	0.012 (2)
C33	0.035 (3)	0.055 (3)	0.067 (3)	0.011 (2)	0.005 (2)	0.010 (2)
C34	0.051 (3)	0.039 (2)	0.055 (3)	0.014 (2)	0.015 (2)	0.001 (2)
C35	0.044 (3)	0.035 (2)	0.036 (2)	0.0047 (18)	0.0039 (18)	−0.0032 (17)
C41	0.0238 (19)	0.0290 (17)	0.0211 (17)	0.0008 (14)	−0.0021 (14)	−0.0029 (14)
C42	0.024 (2)	0.0333 (19)	0.0266 (19)	0.0006 (15)	0.0009 (15)	0.0007 (15)
C43	0.027 (2)	0.050 (2)	0.028 (2)	−0.0006 (18)	0.0044 (16)	−0.0005 (17)
C44	0.028 (2)	0.060 (3)	0.028 (2)	0.0065 (19)	0.0032 (16)	−0.0080 (19)
C45	0.041 (3)	0.040 (2)	0.037 (2)	0.0110 (18)	−0.0002 (18)	−0.0117 (18)
C46	0.042 (2)	0.0311 (19)	0.028 (2)	0.0027 (17)	0.0016 (17)	−0.0041 (16)
C51	0.027 (2)	0.0181 (16)	0.0275 (19)	−0.0019 (14)	0.0017 (15)	−0.0029 (13)
C52	0.028 (2)	0.0332 (19)	0.029 (2)	−0.0045 (16)	−0.0004 (16)	−0.0050 (15)
C53	0.035 (2)	0.043 (2)	0.037 (2)	−0.0061 (18)	−0.0040 (18)	−0.0131 (18)
C54	0.025 (2)	0.035 (2)	0.057 (3)	−0.0069 (17)	0.0002 (18)	−0.0109 (19)
C55	0.031 (2)	0.040 (2)	0.044 (2)	−0.0077 (18)	0.0096 (18)	0.0010 (18)
C56	0.034 (2)	0.037 (2)	0.031 (2)	−0.0071 (17)	0.0044 (17)	−0.0010 (16)
C61	0.026 (2)	0.0261 (17)	0.0256 (18)	0.0020 (15)	−0.0032 (15)	0.0012 (14)
C62	0.029 (2)	0.0288 (18)	0.0195 (18)	−0.0007 (15)	−0.0033 (15)	0.0051 (14)
P3	0.0191 (5)	0.0207 (4)	0.0184 (4)	−0.0002 (3)	0.0024 (3)	−0.0002 (3)
P4	0.0211 (5)	0.0264 (4)	0.0152 (4)	−0.0011 (4)	−0.0001 (3)	−0.0007 (3)
C71	0.0247 (19)	0.0226 (16)	0.0191 (17)	−0.0020 (14)	0.0055 (14)	−0.0007 (13)
C72	0.028 (2)	0.0237 (17)	0.0208 (17)	0.0033 (15)	0.0051 (14)	0.0025 (13)
C73	0.023 (2)	0.037 (2)	0.029 (2)	−0.0033 (16)	0.0066 (15)	0.0008 (16)
C74	0.037 (2)	0.033 (2)	0.031 (2)	−0.0112 (17)	0.0091 (17)	0.0015 (16)
C75	0.040 (2)	0.0245 (18)	0.037 (2)	−0.0011 (17)	0.0070 (18)	0.0071 (16)
C76	0.029 (2)	0.0268 (18)	0.0307 (19)	0.0021 (15)	0.0020 (16)	0.0048 (15)
C81	0.0196 (18)	0.0315 (18)	0.0201 (17)	0.0045 (14)	0.0007 (14)	−0.0002 (14)
C82	0.047 (3)	0.035 (2)	0.033 (2)	0.0011 (18)	0.0119 (19)	−0.0032 (17)
C83	0.064 (3)	0.045 (2)	0.035 (2)	0.016 (2)	0.012 (2)	−0.0095 (19)
C84	0.037 (3)	0.069 (3)	0.031 (2)	0.022 (2)	0.0100 (19)	0.001 (2)
C85	0.035 (3)	0.077 (3)	0.044 (3)	−0.010 (2)	0.020 (2)	−0.005 (2)
C86	0.039 (3)	0.052 (2)	0.045 (3)	−0.012 (2)	0.019 (2)	−0.012 (2)
C91	0.0223 (19)	0.0250 (17)	0.0282 (19)	0.0020 (14)	0.0001 (15)	−0.0078 (14)
C92	0.024 (2)	0.042 (2)	0.036 (2)	0.0008 (17)	0.0035 (16)	−0.0002 (17)
C93	0.031 (2)	0.052 (3)	0.058 (3)	−0.0053 (19)	0.015 (2)	0.005 (2)
C94	0.021 (2)	0.046 (2)	0.080 (4)	−0.0039 (18)	0.003 (2)	−0.011 (2)
C95	0.030 (2)	0.043 (2)	0.052 (3)	0.0058 (19)	−0.0138 (19)	−0.013 (2)
C96	0.030 (2)	0.034 (2)	0.032 (2)	0.0044 (16)	−0.0054 (16)	−0.0035 (16)
C101	0.0238 (19)	0.0367 (19)	0.0169 (17)	0.0055 (15)	0.0001 (14)	0.0027 (14)
C102	0.037 (2)	0.036 (2)	0.0211 (19)	0.0094 (17)	−0.0016 (16)	−0.0006 (15)
C103	0.057 (3)	0.044 (2)	0.027 (2)	0.017 (2)	−0.0007 (19)	−0.0054 (17)
C104	0.065 (3)	0.059 (3)	0.023 (2)	0.024 (2)	0.014 (2)	0.0039 (19)
C105	0.040 (3)	0.070 (3)	0.030 (2)	0.009 (2)	0.0145 (19)	0.015 (2)
C106	0.035 (2)	0.047 (2)	0.0225 (19)	0.0027 (18)	0.0051 (16)	0.0057 (16)
B1	0.090 (5)	0.032 (2)	0.042 (3)	−0.023 (3)	0.015 (3)	−0.006 (2)
F11	0.0766 (19)	0.0377 (12)	0.0389 (13)	−0.0116 (12)	0.0123 (13)	−0.0034 (10)
F12	0.093 (2)	0.0527 (17)	0.083 (2)	0.0031 (16)	0.0340 (19)	−0.0054 (15)
F13	0.112 (3)	0.089 (2)	0.070 (2)	−0.045 (2)	−0.037 (2)	0.0137 (18)
F14	0.247 (5)	0.0607 (19)	0.067 (2)	−0.070 (3)	0.088 (3)	−0.0181 (16)
B2	0.062 (4)	0.056 (3)	0.033 (3)	−0.027 (3)	0.016 (2)	−0.009 (2)
F21	0.071 (2)	0.0651 (18)	0.131 (3)	−0.0384 (16)	0.055 (2)	−0.0372 (19)
F22	0.133 (3)	0.111 (3)	0.0303 (15)	−0.076 (2)	−0.0069 (17)	0.0020 (15)
F23	0.175 (6)	0.090 (4)	0.041 (3)	−0.075 (4)	0.059 (3)	−0.037 (2)
F24	0.102 (5)	0.062 (3)	0.200 (7)	0.015 (3)	−0.088 (4)	−0.012 (4)

### Geometric parameters (Å, °)

**Table:**

Mo1—N1	1.755 (3)	C53—H53	0.9500
Mo1—F1	1.9889 (18)	C54—C55	1.377 (6)
Mo1—P1	2.5032 (9)	C54—H54	0.9500
Mo1—P2	2.5353 (9)	C55—C56	1.398 (6)
Mo1—P3	2.5698 (8)	C55—H55	0.9500
Mo1—P4	2.5754 (9)	C56—H56	0.9500
N1—N2	1.325 (5)	C61—C62	1.526 (5)
N2—H1N2	0.9101	C61—P3	1.848 (4)
N2—H2N2	0.9100	C61—H61A	0.9900
C1—N3	1.464 (4)	C61—H61B	0.9900
C1—P1	1.865 (3)	C62—P4	1.847 (3)
C1—H1A	0.9900	C62—H62A	0.9900
C1—H1B	0.9900	C62—H62B	0.9900
N3—C31	1.358 (5)	P3—C71	1.829 (3)
N3—C2	1.463 (4)	P3—C81	1.832 (3)
C2—P2	1.873 (3)	P4—C91	1.826 (4)
C2—H2A	0.9900	P4—C101	1.833 (3)
C2—H2B	0.9900	C71—C72	1.396 (5)
P1—C11	1.826 (3)	C71—C76	1.402 (5)
P1—C21	1.828 (3)	C72—C73	1.388 (5)
P2—C41	1.827 (3)	C72—H72	0.9500
P2—C51	1.831 (3)	C73—C74	1.382 (5)
C11—C12	1.392 (5)	C73—H73	0.9500
C11—C16	1.396 (5)	C74—C75	1.377 (6)
C12—C13	1.390 (5)	C74—H74	0.9500
C12—H12	0.9500	C75—C76	1.393 (5)
C13—C14	1.377 (6)	C75—H75	0.9500
C13—H13	0.9500	C76—H76	0.9500
C14—C15	1.384 (5)	C81—C82	1.388 (5)
C14—H14	0.9500	C81—C86	1.391 (5)
C15—C16	1.391 (5)	C82—C83	1.395 (6)
C15—H15	0.9500	C82—H82	0.9500
C16—H16	0.9500	C83—C84	1.369 (6)
C21—C26	1.395 (5)	C83—H83	0.9500
C21—C22	1.401 (5)	C84—C85	1.375 (7)
C22—C23	1.388 (5)	C84—H84	0.9500
C22—H22	0.9500	C85—C86	1.394 (6)
C23—C24	1.383 (6)	C85—H85	0.9500
C23—H23	0.9500	C86—H86	0.9500
C24—C25	1.385 (6)	C91—C92	1.389 (5)
C24—H24	0.9500	C91—C96	1.401 (5)
C25—C26	1.389 (5)	C92—C93	1.393 (6)
C25—H25	0.9500	C92—H92	0.9500
C26—H26	0.9500	C93—C94	1.382 (7)
C31—N4	1.363 (5)	C93—H93	0.9500
C31—C35	1.413 (5)	C94—C95	1.372 (7)
N4—C32	1.356 (5)	C94—H94	0.9500
N4—H4	0.8800	C95—C96	1.395 (6)
C32—C33	1.357 (6)	C95—H95	0.9500
C32—H32	0.9500	C96—H96	0.9500
C33—C34	1.391 (7)	C101—C106	1.397 (5)
C33—H33	0.9500	C101—C102	1.398 (5)
C34—C35	1.372 (6)	C102—C103	1.392 (5)
C34—H34	0.9500	C102—H102	0.9500
C35—H35	0.9500	C103—C104	1.374 (7)
C41—C42	1.385 (5)	C103—H103	0.9500
C41—C46	1.401 (5)	C104—C105	1.383 (7)
C42—C43	1.396 (5)	C104—H104	0.9500
C42—H42	0.9500	C105—C106	1.393 (6)
C43—C44	1.374 (6)	C105—H105	0.9500
C43—H43	0.9500	C106—H106	0.9500
C44—C45	1.383 (6)	B1—F14	1.358 (6)
C44—H44	0.9500	B1—F13	1.359 (7)
C45—C46	1.397 (5)	B1—F12	1.397 (7)
C45—H45	0.9500	B1—F11	1.407 (6)
C46—H46	0.9500	B2—F24'	1.241 (10)
C51—C52	1.393 (5)	B2—F22	1.322 (5)
C51—C56	1.394 (5)	B2—F23	1.330 (6)
C52—C53	1.391 (5)	B2—F21	1.348 (5)
C52—H52	0.9500	B2—F24	1.475 (7)
C53—C54	1.384 (6)	B2—F23'	1.570 (10)
			
N1—Mo1—F1	178.70 (11)	C55—C54—C53	119.8 (4)
N1—Mo1—P1	86.88 (10)	C55—C54—H54	120.1
F1—Mo1—P1	91.94 (6)	C53—C54—H54	120.1
N1—Mo1—P2	86.22 (9)	C54—C55—C56	120.4 (4)
F1—Mo1—P2	94.33 (6)	C54—C55—H55	119.8
P1—Mo1—P2	89.65 (3)	C56—C55—H55	119.8
N1—Mo1—P3	94.46 (9)	C51—C56—C55	120.5 (4)
F1—Mo1—P3	85.10 (5)	C51—C56—H56	119.8
P1—Mo1—P3	95.38 (3)	C55—C56—H56	119.8
P2—Mo1—P3	174.95 (3)	C62—C61—P3	110.1 (2)
N1—Mo1—P4	101.62 (10)	C62—C61—H61A	109.6
F1—Mo1—P4	79.52 (6)	P3—C61—H61A	109.6
P1—Mo1—P4	170.35 (3)	C62—C61—H61B	109.6
P2—Mo1—P4	95.42 (3)	P3—C61—H61B	109.6
P3—Mo1—P4	79.53 (3)	H61A—C61—H61B	108.1
N2—N1—Mo1	177.1 (3)	C61—C62—P4	109.7 (2)
N1—N2—H1N2	115.4	C61—C62—H62A	109.7
N1—N2—H2N2	125.8	P4—C62—H62A	109.7
H1N2—N2—H2N2	118.7	C61—C62—H62B	109.7
N3—C1—P1	110.7 (2)	P4—C62—H62B	109.7
N3—C1—H1A	109.5	H62A—C62—H62B	108.2
P1—C1—H1A	109.5	C71—P3—C81	105.51 (15)
N3—C1—H1B	109.5	C71—P3—C61	104.13 (16)
P1—C1—H1B	109.5	C81—P3—C61	100.27 (16)
H1A—C1—H1B	108.1	C71—P3—Mo1	116.78 (11)
C31—N3—C2	121.6 (3)	C81—P3—Mo1	121.04 (11)
C31—N3—C1	123.4 (3)	C61—P3—Mo1	106.61 (11)
C2—N3—C1	114.7 (3)	C91—P4—C101	104.34 (16)
N3—C2—P2	115.4 (2)	C91—P4—C62	102.59 (16)
N3—C2—H2A	108.4	C101—P4—C62	101.48 (16)
P2—C2—H2A	108.4	C91—P4—Mo1	120.50 (12)
N3—C2—H2B	108.4	C101—P4—Mo1	119.52 (12)
P2—C2—H2B	108.4	C62—P4—Mo1	105.48 (11)
H2A—C2—H2B	107.5	C72—C71—C76	118.6 (3)
C11—P1—C21	106.26 (15)	C72—C71—P3	119.3 (2)
C11—P1—C1	100.35 (15)	C76—C71—P3	122.1 (3)
C21—P1—C1	99.87 (15)	C73—C72—C71	120.6 (3)
C11—P1—Mo1	114.87 (11)	C73—C72—H72	119.7
C21—P1—Mo1	119.87 (11)	C71—C72—H72	119.7
C1—P1—Mo1	112.95 (11)	C74—C73—C72	120.6 (4)
C41—P2—C51	103.36 (15)	C74—C73—H73	119.7
C41—P2—C2	102.80 (16)	C72—C73—H73	119.7
C51—P2—C2	99.92 (16)	C75—C74—C73	119.4 (3)
C41—P2—Mo1	122.48 (11)	C75—C74—H74	120.3
C51—P2—Mo1	110.44 (11)	C73—C74—H74	120.3
C2—P2—Mo1	114.99 (11)	C74—C75—C76	121.0 (3)
C12—C11—C16	118.8 (3)	C74—C75—H75	119.5
C12—C11—P1	120.0 (3)	C76—C75—H75	119.5
C16—C11—P1	120.8 (2)	C75—C76—C71	119.9 (3)
C13—C12—C11	120.2 (3)	C75—C76—H76	120.1
C13—C12—H12	119.9	C71—C76—H76	120.1
C11—C12—H12	119.9	C82—C81—C86	118.3 (3)
C14—C13—C12	120.7 (3)	C82—C81—P3	123.3 (3)
C14—C13—H13	119.7	C86—C81—P3	118.4 (3)
C12—C13—H13	119.7	C81—C82—C83	120.4 (4)
C13—C14—C15	119.7 (3)	C81—C82—H82	119.8
C13—C14—H14	120.1	C83—C82—H82	119.8
C15—C14—H14	120.1	C84—C83—C82	120.8 (4)
C14—C15—C16	120.0 (3)	C84—C83—H83	119.6
C14—C15—H15	120.0	C82—C83—H83	119.6
C16—C15—H15	120.0	C83—C84—C85	119.4 (4)
C15—C16—C11	120.6 (3)	C83—C84—H84	120.3
C15—C16—H16	119.7	C85—C84—H84	120.3
C11—C16—H16	119.7	C84—C85—C86	120.5 (4)
C26—C21—C22	119.1 (3)	C84—C85—H85	119.8
C26—C21—P1	118.7 (3)	C86—C85—H85	119.8
C22—C21—P1	122.0 (3)	C81—C86—C85	120.5 (4)
C23—C22—C21	120.4 (3)	C81—C86—H86	119.7
C23—C22—H22	119.8	C85—C86—H86	119.7
C21—C22—H22	119.8	C92—C91—C96	118.9 (3)
C24—C23—C22	120.1 (4)	C92—C91—P4	119.6 (3)
C24—C23—H23	119.9	C96—C91—P4	121.5 (3)
C22—C23—H23	119.9	C91—C92—C93	120.2 (4)
C23—C24—C25	119.8 (4)	C91—C92—H92	119.9
C23—C24—H24	120.1	C93—C92—H92	119.9
C25—C24—H24	120.1	C94—C93—C92	120.6 (4)
C24—C25—C26	120.7 (4)	C94—C93—H93	119.7
C24—C25—H25	119.6	C92—C93—H93	119.7
C26—C25—H25	119.6	C95—C94—C93	119.6 (4)
C25—C26—C21	119.9 (3)	C95—C94—H94	120.2
C25—C26—H26	120.1	C93—C94—H94	120.2
C21—C26—H26	120.1	C94—C95—C96	120.6 (4)
N3—C31—N4	120.0 (3)	C94—C95—H95	119.7
N3—C31—C35	123.8 (3)	C96—C95—H95	119.7
N4—C31—C35	116.2 (3)	C95—C96—C91	120.0 (4)
C32—N4—C31	123.9 (4)	C95—C96—H96	120.0
C32—N4—H4	118.0	C91—C96—H96	120.0
C31—N4—H4	118.0	C106—C101—C102	119.1 (3)
N4—C32—C33	120.4 (4)	C106—C101—P4	118.5 (3)
N4—C32—H32	119.8	C102—C101—P4	122.3 (3)
C33—C32—H32	119.8	C103—C102—C101	120.2 (4)
C32—C33—C34	118.0 (4)	C103—C102—H102	119.9
C32—C33—H33	121.0	C101—C102—H102	119.9
C34—C33—H33	121.0	C104—C103—C102	120.4 (4)
C35—C34—C33	121.6 (4)	C104—C103—H103	119.8
C35—C34—H34	119.2	C102—C103—H103	119.8
C33—C34—H34	119.2	C103—C104—C105	119.9 (4)
C34—C35—C31	119.9 (4)	C103—C104—H104	120.1
C34—C35—H35	120.1	C105—C104—H104	120.1
C31—C35—H35	120.1	C104—C105—C106	120.7 (4)
C42—C41—C46	119.4 (3)	C104—C105—H105	119.7
C42—C41—P2	120.3 (3)	C106—C105—H105	119.7
C46—C41—P2	120.3 (3)	C105—C106—C101	119.7 (4)
C41—C42—C43	120.2 (3)	C105—C106—H106	120.1
C41—C42—H42	119.9	C101—C106—H106	120.1
C43—C42—H42	119.9	F14—B1—F13	111.3 (5)
C44—C43—C42	120.2 (4)	F14—B1—F12	110.2 (5)
C44—C43—H43	119.9	F13—B1—F12	107.7 (4)
C42—C43—H43	119.9	F14—B1—F11	110.0 (4)
C43—C44—C45	120.3 (4)	F13—B1—F11	110.5 (5)
C43—C44—H44	119.8	F12—B1—F11	107.1 (4)
C45—C44—H44	119.8	F24'—B2—F22	129.4 (8)
C44—C45—C46	120.0 (4)	F24'—B2—F23	47.9 (8)
C44—C45—H45	120.0	F22—B2—F23	117.8 (4)
C46—C45—H45	120.0	F24'—B2—F21	115.8 (8)
C45—C46—C41	119.8 (4)	F22—B2—F21	111.7 (4)
C45—C46—H46	120.1	F23—B2—F21	119.8 (4)
C41—C46—H46	120.1	F24'—B2—F24	57.5 (8)
C52—C51—C56	118.2 (3)	F22—B2—F24	92.9 (5)
C52—C51—P2	118.5 (3)	F23—B2—F24	102.6 (5)
C56—C51—P2	123.0 (3)	F21—B2—F24	107.0 (4)
C53—C52—C51	121.1 (4)	F24'—B2—F23'	104.1 (9)
C53—C52—H52	119.4	F22—B2—F23'	83.9 (6)
C51—C52—H52	119.4	F23—B2—F23'	56.2 (5)
C54—C53—C52	120.0 (4)	F21—B2—F23'	99.8 (6)
C54—C53—H53	120.0	F24—B2—F23'	152.2 (6)
C52—C53—H53	120.0		
